# Honokiol-loaded nanoparticles for targeted bacterial eradication and treatment of MRSA infection

**DOI:** 10.1016/j.mtbio.2025.102562

**Published:** 2025-11-17

**Authors:** Shouli Yi, Hongjuan Zhang, Zhen Yang, Di Wu, Dan Shao, Jiongjie He, Yu Liu, Baocheng Hao, Shengyi Wang

**Affiliations:** a Key Laboratory of New Animal Drug Project, Gansu Province/Key Laboratory of Veterinary Pharmaceutical Development, Ministry of Agriculture and Rural Affairs/Lanzhou Institute of Husbandry and Pharmaceutical Sciences of Chinese Academy of Agricultural Sciences, Lanzhou, 730050, PR China; b College of Animal Science and Technology, Guangxi University, Nanning, 530005, PR China

**Keywords:** Honokiol, Rhamnolipid, pH-responsive, Anti-MRSA, Biofilm

## Abstract

Methicillin-resistant *Staphylococcus aureus* (MRSA) infections and the formation of associated biofilms often lead to refractory and recurrent bacterial infections, posing a serious threat to public health. Herein, a pH-responsive antibiotic-free nanoparticle (HNK@RHL-COS NPs) was prepared by encapsulating the natural antimicrobial agent honokiol (HNK) with rhamnolipid-modified chitosan, enabling targeted disassembling at the acidic infection site, destruction of the biofilm by rhamnolipid, thereby enhancing the penetration of honokiol and chitosan, thus achieving their synergistic bactericidal effect. HNK@RHL-COS NPs exhibited enhanced antimicrobial effects against MRSA, with a minimum inhibitory concentration (MIC) value of 2 μg/mL, significantly inhibited the formation of biofilm and eradicated the mature biofilm. Furthermore, HNK@RHL-COS NPs efficiently accelerated the healing of wounds in MRSA-infected mice, alleviated bacterial peritonitis in mice, and demonstrated excellent biocompatibility *in vitro* and *in vivo*. Collectively, this study provides a novel and safety solution for the clinical treatment of MRSA infections.

## Highlights

• HNK@RHL-COS NPS shows features that respond to an acidic infection environment.
• HNK@RHL-COS NPS can inhibit MRSA and its biofilm infection at low concentrations.
• HNK@RHL-COS NPS can relieve MRSA-infected skin wounds and promote wound healing.

## Graphical abstract

**Figure:**
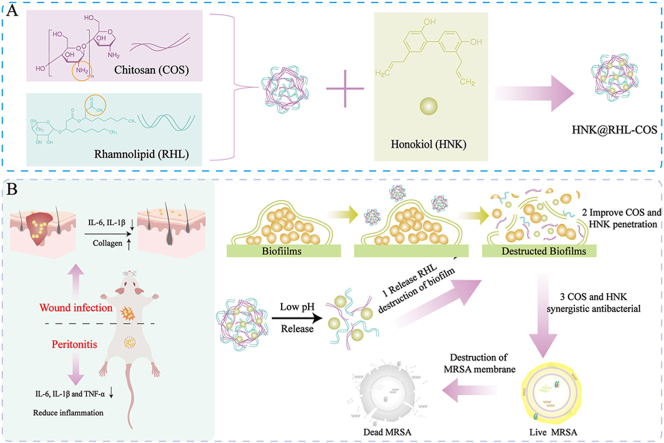

## 1 Introduction

Bacterial infections are prevalent and can lead to various symptoms and complications, posing a serious threat to global public health. The misuse and overuse of antibiotics have led to the ongoing emergence of multidrug-resistant strains, among which methicillin-resistant *Staphylococcus aureus* (MRSA), with multidrug resistance and high pathogenicity, is the leading pathogen responsible for triggering skin infections, pneumonia, sepsis, and hospital-acquired infections [1]. Seriously, MRSA can easily form biofilm, a 3D bacterial community created by bacteria embedding themselves in extracellular polymeric substances (EPS) secreted by themselves, contributing to exacerbated bacterial resistance [2]. Biofilm formation not only limits the penetration of antibiotics, allowing bacteria to evade the host immune response, but also leads to recurrent infections through the continuous release of planktonic bacteria, presenting a huge challenge to the clinical treatment of MRSA [[3], [4], [5], [6]]. Furthermore, the development of new antibiotics lags far behind the development of bacterial resistance. Therefore, it is crucial to develop new treatment strategies to eradicate MRSA and its associated biofilms effectively.

Recently, plant-derived natural antimicrobial agents have received widespread attention as a strategy for MRSA control due to their multiple antimicrobial mechanisms and low risk of drug resistance development [7]. Furthermore, many of these agents exhibit anti-inflammatory and antioxidant properties, thus enhancing their effectiveness in treating bacterial infections. Honokiol (HNK), a bisphenol compound extracted from *Magnolia officinalis,* exhibits potent anti-MRSA activity [[8], [9], [10], [11]]. Mechanically, HNK inhibits biofilm formation by suppressing polysaccharide intercellular adhesion (PIA) and the production of extracellular polymeric substances [12]; it also exerts broad-spectrum anti-inflammatory effects via the NF-κB pathway and downregulation of key pro-inflammatory mediators [13]. However, after long-term intake of HNK extract in mice, it accumulates in the serum, urine, and kidneys, leading to abnormal renal function markers and ultrastructural changes, and may cause allergic contact dermatitis. *In vitro* studies have demonstrated that HNK can influence specific enzyme activities, and there is a potential risk of interaction with other medications or substances [14]. Moreover, the poor water solubility, instability, and low bioavailability of HNK hinder its clinical application [15].

Over the last several decades, nanoparticle drug delivery systems (NDDS) have been considered a promising strategy to develop antimicrobial agents since they can improve the solubility, stability, absorption, and bioavailability of the drug while also targeting the transport of the drug to the disease site [16,17]. Many natural antimicrobial agents, such as rhein, berberine and chlorogenic acid, have been successfully developed into nanoparticles with improved antimicrobial efficacy [18,19]. In particular, bacterial microenvironment-responsive nanoparticles (pH-responsive, redox-responsive and enzyme-responsive), without additional external stimuli, have received extensive attention owing to their targeted delivery of drugs to the infection site and the potential of binding to the bacterial cell wall. However, the application and commercialization of synthetic stimuli-responsive nanomaterials are restricted due to their poor biocompatibility and harsh synthesis conditions. Therefore, natural antimicrobial nanomaterials have drawn more attention. Chitosan (COS), a biocompatible natural cationic polysaccharide, displays good antimicrobial [20,21] and anti-inflammatory properties and has been widely used in drug delivery systems [22,23]. Studies demonstrated that rhamnolipids (RHL), an anionic biosurfactant, possess excellent anti-biofilm activity, which destroys biofilms of MRSA by disrupting EPS of biofilm and inhibiting the reattachment of planktonic bacteria, thereby promoting the penetration of antimicrobial agents [[24], [25], [26], [27], [28]]. Research has shown that the combination of RHL and COS enhances its effectiveness against bacteria [29]. Inspired by this, HNK-loaded rhamnolipid-modified chitosan nanoparticles could potentially eradicate MRSA and its associated biofilm.

**Scheme 1:**
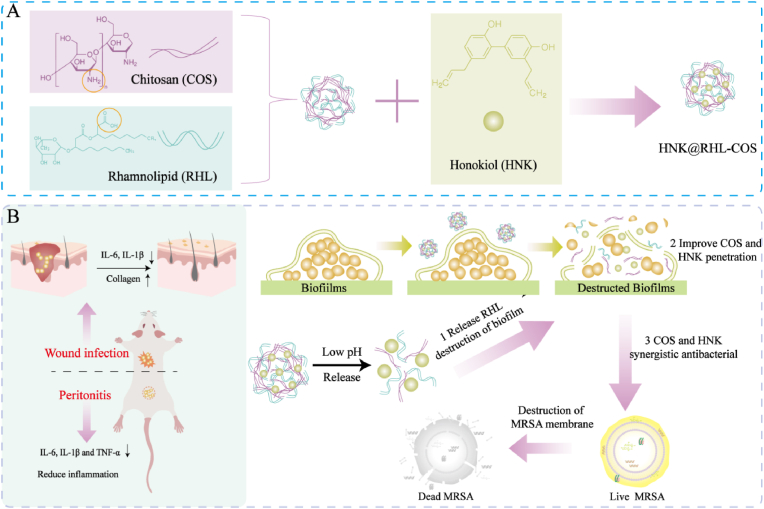
Fabrication and application of HNK@RHL-COS NPs. (A) The preparation of HNK@RHL-COS NPs. (B) The application of HNK@RHL-COS NPs in MRSA biofilm eradication.

Herein, we developed pH-responsive antibiotic-free nanoparticles for targeted bacterial eradication and enhanced MRSA biofilm penetration (Scheme 1A). Specifically, rhamnolipid-modified chitosan nanoparticles were utilized as a carrier, and the natural antimicrobial compound honokiol was loaded (HNK@RHL-COS NPs). HNK@RHL-COS NPs disassembled in the low pH microenvironment of the infection site due to the breakdown of the amide bond formed by the amino group of chitosan and the carboxyl group of rhamnolipid. Then, the released rhamnolipid disrupted biofilms by dispersing extracellular polysaccharides and proteins, which enhanced the penetration of the natural antimicrobial agents honokiol and chitosan, thereby achieving their synergistic killing of MRSA (Scheme 1B). Studies indicated that HNK@RHL-COS NPs exhibited superior antimicrobial properties against MRSA, effectively inhibited biofilm formation and destroyed established biofilms. In addition, HNK@RHL-COS NPs demonstrated significant therapeutic effects in a mouse skin infection model and a bacterial peritonitis model. This work sheds light on a promising antimicrobial strategy for the effective treatment of MRSA infection.

## 2 Materials and methods

### 2.1 Materials

Honokiol and rhamnolipid (90 %) were purchased from Shanghai Macklin Biochemical Technology Co., Ltd. (Shanghai, China). Chitosan (degree of deacetylation ≥95 %) and glutaraldehyde solution were obtained from Shanghai Aladdin Biochemical Technology Co., Ltd. (Shanghai, China). L-929 cells (NCTC clone 929), crystal violet, 4, 6-diamidino-2-phenylindole (DAPI), propidium iodide (PI), Bacterial/yeast viability staining kit, BCA protein assay kit and CCK8 kit were acquired from Wuhan Servicebio Technology Co., Ltd. (Wuhan, China). MRSA (ATCC 43300) was bought from the American Type Culture Collection (Manassas, Virginia, USA). Calcein-AM/PI, Live/Dead Cell Double Stain Kit was acquired from Beijing Solarbio Science & Technology Co., Ltd. (Beijing, China). Sulfo-Cyanine 5.5 (Cy5.5) was obtained from MedChemExpress (New Jersey, USA).

### 2.2 Preparation of HNK@RHL-COS NPs

RHL-COS NPs and HNK@RHL-COS NPs were prepared with reference to the reported method with a slight modification [30]. Briefly, 1 mL of sodium tripolyphosphate (TPP) and 2.5 mL of rhamnolipid (RHL, 1 mg/mL) were sequentially dripped into 5 mL of chitosan solution (0.1 mg/mL) and stirred overnight. Next, 100 μL of glutaraldehyde (GA, 0.5 wt%) was dripped into the mixed solution at the same rate. After stirring for 12 h, the obtained solution was dialyzed (pH 7.4 phosphate buffer, 24 h) to obtain RHL-COS NPs. HNK@RHL-COS NPs were prepared using the same procedure, except that honokiol (2 mg/mL) was added and stirred for 24 h before dialysis.

### 2.3 Characterization of HNK@RHL-COS NPs

To confirm the successful fabrication of HNK@RHL-COS NPs, we conducted a systematic characterization. The drug loading and encapsulation efficiency of HNK@RHL-COS NPs were determined by HPLC (Waters, E2695, USA). The particle size and zeta potential of HNK@RHL-COS NPs were determined using Zetasizer (Malvern, Nano ZS90, UK). The morphology of HNK@RHL-COS NPs was observed by Transmission Electron Microscope (TEM, FEI Talos F200x, USA). The Fourier Transform infrared spectra (FT-IR) of HNK@RHL-COS NPs and RHL-COS NPs were obtained using a spectrometer (Thermo Fisher Scientific, Nicolet iS20, USA). Raman spectra analysis of HNK@RHL-COS NPs and RHL-COS NPs was performed using a Raman spectrometer (LabRam HR Evolution, HORIBA Scientific, FRA). The stability of HNK@RHL-COS NPs was evaluated by measuring the particle size on days 7, 14, 21 and 28 at 4 °C and room temperature, as well as the particle size at 0, 6, 12 and 24 h in PBS containing 50 % FBS at 37 °C.

### 2.4 Acidic microenvironment-responsive release

HNK@RHL-COS NPs (3 mL) were placed into a dialysis bag and immersed in a release medium (PBS with pH 5.5/7.4), then incubated in a shaking incubator (37 °C, 100 rpm). At 0, 1, 2, 3, 4, 5, 6, 8, 10, 12, 24 and 48 h, 2 mL of the release medium was withdrawn and replaced with an equal amount of fresh release medium. The concentration of HNK was measured by HPLC to determine the cumulative release rate of HNK@RHL-COS NPs. Finally, after 48 h, the remaining samples were collected and freeze-dried for FT-IR analysis (Thermo Fisher Scientific, Nicolet iS20, USA) and Raman spectra analysis (LabRam HR Evolution, HORIBA Scientific, FRA).

### 2.5 In vitro antimicrobial activity of HNK@RHL-COS NPs

#### 2.5.1 Minimal inhibitory concentration (MIC) and minimum bactericidal concentration (MBC)

The MIC and MBC of HNK@RHL-COS NPs were evaluated using a broth microdilution assay and a plate counting method, respectively [31]. Briefly, bacterial suspensions were adjusted to 1 × 10^6^ CFU/mL in Mueller-Hinton (MH) broth medium. Serial two-fold dilutions of HNK@RHL-COS NPs (32 μg/mL), RHL-COS NPs (80 μg/mL), and free HNK (128 μg/mL) were dispensed into 96-well plates, followed by inoculation with the standardized bacterial suspension. After 24 h of incubation at 37 °C, the MIC was recorded as the lowest concentration showing no visible bacterial growth. To determine the MBC, 100 μL aliquots from wells with no visible bacterial growth were subcultured on MH agar plates and incubated overnight at 37 °C. The MBC was defined as the lowest concentration achieving ≥99.9 % reduction of the initial inoculum, corresponding to a colony count of less than 1 × 10^2^ CFU/mL.

#### 2.5.2 Killing curve

HNK@RHL-COS NPs, RHL-COS NPs and HNK at different concentrations were separately added to bacterial culture tubes containing MRSA (1 × 10^6^ CFU/mL). Samples were incubated at 37 °C for 24 h (160 rpm) and were taken at 1 h intervals to determine OD_600_ values and to plot the killing curve. In addition, samples were diluted and plated onto MH agar plates at 0, 2, 4, 8, 12 and 24 h to count the CFU numbers.

#### 2.5.3 Bacterial live/dead staining

HNK@RHL-COS NPs, RHL-COS NPs and HNK at different concentrations were separately added to bacterial culture tubes containing MRSA (1 × 10^6^ CFU/mL). Samples were incubated at 37 °C for 12 h (160 rpm). SYTO-9 and PI were added for staining, and CLSM (ZEISS LSM800, ZEISS, DE) was used to observe the bacteria.

### 2.6 Anti-biofilm effect of HNK@RHL-COS NPs

#### 2.6.1 The effect of HNK@RHL-COS NPs on the formation of biofilm

MRSA (1 × 10^6^ CFU/mL) and different concentrations of drugs were added to 96-well plates. After 12 h of culture, the supernatant was slowly removed and washed with PBS. 4 % paraformaldehyde was added to each well for 30 min to fix the biofilm. After washing with PBS, 1 % crystal violet was added and incubated for 15 min. Finally, crystal violet was dissolved in 33 % acetic acid, and the absorbance at 570 nm was determined.

#### 2.6.2 The effect of HNK@RHL-COS NPs on the mature biofilms

MRSA (1 × 10^6^ CFU/mL, 100 μL) was added to 96-well plates, and the supernatant was slowly removed after incubation for 24 h. Then, HNK@RHL-COS NPs, RHL-COS NPs and HNK at various concentrations were separately added, and the supernatant was slowly removed after 12 h of incubation. Crystal violet staining was performed and the OD_570_ values were determined using a spectrophotometer [32].

### 2.7 Bacterial morphology and cell membrane permeability

#### 2.7.1 Scanning electron microscope

HNK@RHL-COS NPs, RHL-COS NPs and HNK at various concentrations were added to tubes containing MRSA (1 × 10^6^ CFU/mL). After incubation at 37 °C for 24 h, samples were centrifuged and rinsed three times with PBS, then fixed overnight with 2.5 % glutaraldehyde. Following gradient dehydration, freeze-drying and gold spraying, the bacterial morphology was observed using SEM.

#### 2.7.2 Regulation of cell membrane permeability

Various concentrations of HNK@RHL-COS NPs, RHL-COS NPs and HNK, along with resuspended MRSA (1 × 10^6^ CFU/mL) in PBS, were added to a black 96-well plate and cultured for 12 h. Fluorescent dye PI was added to each to achieve a final concentration of 7.5 μg/mL. After incubation in the dark for 30 min, the fluorescence intensity in each well was measured at an excitation wavelength of 535 nm and an emission wavelength of 615 nm.

#### 2.7.3 Intracellular material leakage

The resuspended MRSA (1 × 10^6^ CFU/mL) in PBS was co-cultured with different concentrations of HNK@RHL-COS NPs, RHL-COS NPs and HNK for 12 h. After centrifugation, the supernatant was collected for use, and the bacterial precipitate was disrupted ultrasonically. The protein content in both the supernatant and precipitate was determined using a BCA kit.

After treating the MRSA in the same way, the supernatant was added to black 96-well plates, and DAPI was added to achieve a final concentration of 0.5 μg/mL. The mixture was incubated in the dark for 30 min, and the fluorescence intensity of DNA and RNA was measured at excitation wavelengths of 364 nm and 400 nm, respectively.

The content of polysaccharides in the supernatant and precipitate was determined using the phenol-sulfuric acid method. Briefly, after treating the MRSA in the same way, 5 % phenol (1 mL) and sulfuric acid (5 mL) were added to the supernatant. Following the reaction in a water bath at 90 °C for 1 h, the mixture was cooled for 20 min, and the OD_490_ was determined using a spectrophotometer. The content of polysaccharides in the precipitate was determined using the same method after ultrasonic fragmentation.

### 2.8 Cell migration assay

L929 cells were seeded in a 6-well plate at a density of 5 × 10^5^ cells/mL, and the original medium was discarded after the confluency was greater than 95 %. The scratches with uniform thickness were created at the bottom of the 6-well plate. After washing, the wells were treated with medium containing HNK@RHL-COS NPs, RHL-COS NPs, HNK or only medium (control) (serum <5 %). The migration of L929 cells was photographed and recorded at 12 h, 24 h and 48 h after administration [33].

### 2.9 Anti-inflammatory effect *in vitro*

RAW 264.7 cells (5 × 10^5^ cells/mL) were seeded in 6-well plates and incubated for 12 h; then, LPS was added to achieve a final concentration of 1 μg/mL. After 24 h of stimulation, HNK@RHL-COS NPs, RHL-COS NPs and HNK were added and incubated for 24 h. After that, the supernatants of different groups were collected, and the levels of NO were detected using the Griess method, while the contents of TNF-α, IL-6 and IL-1β were measured using the ELISA kits [34].

### 2.10 Antimicrobial test *in vivo*

BALB/c mice (female, 18–22 g) were purchased from the Lanzhou Veterinary Research Institute of Chinese Academy of Agricultural Sciences (SCXK(Gan)2020-0002). All animal experiments adhered to the ARRIVE standards and received approval from the Ethics Committee of the Lanzhou Institute of Husbandry and Pharmaceutical Sciences, part of the Chinese Academy of Agricultural Sciences (No. 2024–57).

#### 2.10.1 Mouse skin infection model

Thirty mice were randomly divided into five groups (Control group, Model group, HNK@RHL-COS group, RHL-COS group and HNK group), with 6 mice in each group. The mouse skin infection model was established according to the reported method [35,36]. After anesthesia with 0.5 % pentobarbital sodium, the model was prepared by removing skin hair from their backs, disinfecting the area, and injecting them with 100 μL of MRSA (1 × 10^6^ CFU/mL), while the control group received an injection of PBS. Following 24 h of infection, the mice were injected in situ with 100 μL of HNK (16 μg/mL), RHL-COS NPs, and HNK@RHL-COS NPs (containing an equivalent amount of HNK at 16 μg/mL), respectively. The mice in the model group and the control group were treated with PBS. During the treatment, the apparent morphology, area size, and bacterial load of the wound area were recorded daily. At the end of the treatment, the mice were euthanized, and ELISA was used to determine the inflammatory factors (TNF-α, IL-1β, IL-6, and NO) in the serum of the mice. The skin from the infected site was collected and homogenized in sterile PBS, followed by a plate colony count after dilution. Additionally, another portion of the skin was fixed in 4 % paraformaldehyde for H&E and Masson staining.

#### 2.10.2 Mouse bacterial peritonitis model

Subsequently, an MRSA bacterial peritonitis model was established [37]. Briefly, thirty female mice were randomly divided into five groups (Control group, Model group, HNK@RHL-COS group, RHL-COS group and HNK group) with six mice in each group. Except for the Control group, the mice in the other groups were intraperitoneally injected with MRSA suspension (100 μL, 1 × 10^6^ CFU/mL). After infection for 2 h, the mice were treated via the tail vein with 100 μL of HNK (16 μg/mL), RHL-COS NPs, and HNK@RHL-COS NPs (containing an equivalent amount of HNK at 16 μg/mL), respectively. The mice in the model and control groups were treated with PBS. After 48 h of treatment, mice were intraperitoneally injected with 2 mL of sterile PBS and then euthanized. The serum was collected for detecting inflammatory factors (TNF-α, IL-1β, IL-6, and NO), and the ascites was collected for bacterial counting. The recovered ascites was divided into three parts: the first sample was diluted and plated on agar for total bacterial counts, the second was centrifuged to collect the supernatant for extracellular bacterial counts, and the last was lysed with Triton X-100 after removing extracellular bacteria to count intracellular bacteria. Finally, the heart, liver, spleen, lungs, and kidneys were fixed and examined with H&E staining.

Cy5.5@RHL-COS NPs were prepared by replacing HNK with Cy5.5, following the aforementioned method. Subsequently, Cy5.5@RHL-COS NPs were administered via tail vein injection to healthy mice and mice infected with MRSA via intraperitoneal injection 2 h prior. Fluorescence distribution *in vivo* was observed and recorded using an *in vivo* optical imaging system (DPM, DPM-IVFM-NIR-II, China) at 1, 2, 4, 6, 12, and 24 h post-injection [38].

### 2.11 Biocompatibility evaluation

#### 2.11.1 Hemolysis assay

First, mouse red blood cells were diluted to a concentration of 4 % using PBS. Next, 500 μL of HNK@RHL-COS NPs, RHL-COS NPs and HNK with various concentrations were added. 0.5 % Triton X-100 and PBS were added to the control group and the blank group, respectively. After incubation at 37 °C for 2 h, samples were centrifuged and the supernatant was transferred to 96-well plates. The OD_540_ values were measured and the hemolysis rate was calculated according to the following formula.

$$\text{Hemolysis}\;\text{Rate}\;\left(\%\right)=\frac{{\text{OD}}_{\text{Sample}}‐{\text{OD}}_{\text{Blank}}}{{\text{OD}}_{\text{Control}}‐{\text{OD}}_{\text{Blank}}}\times 100\%$$

#### 2.11.2 CCK8 assay

The L929 cells were seeded in 96-well plates at a density of 1 × 10^4^/well and cultured overnight. Various concentrations of HNK@RHL-COS NPs, RHL-COS NPs and HNK were added to each well. After incubation for 6, 12 and 24 h, respectively, 100 μL of CCK-8 (diluted 10 times with culture medium) was added to each well and incubated for 1 h. Finally, OD_450_ values were measured using a microplate reader.

#### 2.11.3 In vivo toxicity

Mice were randomly divided into four groups (Control group, HNK@RHL-COS group, RHL-COS group and HNK group), with 6 mice in each group. During intraperitoneal administration, the body weight of the mice was recorded daily. After 7 days of continuous administration, the mice were euthanized, and blood samples were collected for routine blood tests. The primary organs (heart, liver, spleen, lung, and kidney) of the mice were collected for H&E staining.

#### 2.11.4 Cell live/dead staining

L929 cells were seeded in 6-well plates at a density of 5 × 10^5^ cells/mL and cultured until reaching >95 % confluency. The cells were then treated with medium containing HNK@RHL-COS NPs, RHL-COS NPs, or HNK for 12 h. Subsequently, cells were harvested and washed twice with 1 × assay buffer and co-stained with Calcein-AM and PI. Fluorescence imaging was performed using a ZERO fluorescence cell imaging system (LeiTe Technology, China).

### 2.12 Statistical analysis

All values are reported as mean ± standard deviation (SD). GraphPad Prism 10.0 was used for statistical analysis of the mean, and the differences between groups were tested with one-way analysis of variance. Statistical significance was set at *p* < 0.05, with ∗ or ^#^ indicating *p* < 0.05, ∗∗ or ^##^ indicating *p* < 0.01, and ∗∗∗ or ^###^ indicating *p* < 0.001.

## 3 Results and discussion

### 3.1 Preparation and characterization of HNK@RHL-COS NPs

**Fig. 1:**
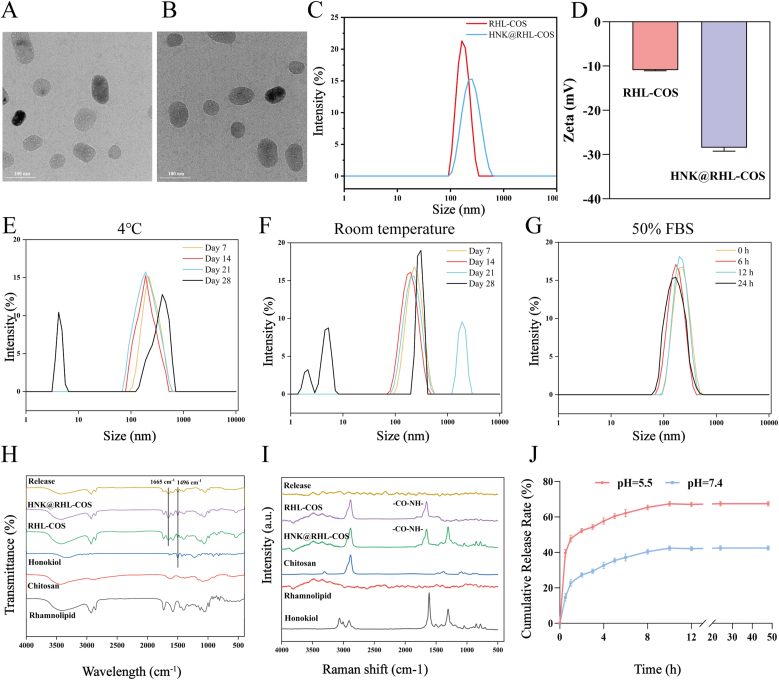
Construction and characterization of HNK@RHL-COS NPs. (A) TEM images of RHL-COS NPs. (B) TEM images of HNK@RHL-COS NPs. (C) Size distribution of HNK@RHL-COS NPs and RHL-COS NPs. (D) The zeta potential of HNK@RHL-COS NPs and RHL-COS NPs. (E, F and G) The stability of HNK@RHL-COS NPs at 4 °C, room temperature and 50 % FBS conditions. (H) FT-IR spectrum results. (I) Raman Spectrum results. (J) Release profiles of HNK from HNK@RHL-COS NPs under different conditions.

HNK@RHL-COS was prepared using a combined ionic crosslinking and chemical crosslinking method [39,40]. The preliminary experiment identified the optimal formulation with an encapsulation efficiency of 96.5 % and a drug loading of 1.9 % (COS: TPP = 5:1, COS: RHL = 2:1, COS: GA = 50:1, RHL-COS: HNK = 10:1) (Fig. S1–S2). As observed by TEM, both RHL-COS NPs and HNK@RHL-COS NPs displayed a uniform spherical shape, with HNK@RHL-COS NPs showing a slightly larger diameter (Fig. 1A and B). Dynamic Light Scattering (DLS) analysis confirmed that the particle size of RHL-COS NPs was about 163 ± 32 nm. After loading HNK, the particle size of HNK@RHL-COS NPs slightly increased to 194 ± 45 nm (PDI = 0.117 ± 0.027), which aligns with TEM results (Fig. 1C). It was also found that the zeta potential of NPs changed from −10.9 mV to −28.5 mV after HNK loading (Fig. 1D). The particle size of HNK@RHL-COS NPs remained stable over 14 days at 4 °C and room temperature, with noticeable aggregation and dissociation at the 28th and 21st days, respectively, indicating good stability of HNK@RHL-COS NPs (Fig. 1E and F). Moreover, HNK@RHL-COS NPs were incubated with PBS containing 50 % FBS at 37 °C, and no obvious changes were observed in their particle size within 24 h, indicating that HNK@RHL-COS NPs may not be affected by various proteins in the blood during systemic circulation, which is beneficial for achieving their targeted therapy (Fig. 1G).

In addition, the FTIR spectra of RHL-COS NPs and HNK@RHL-COS NPs showed obvious bending vibration peaks at 1665 cm^−1^, indicating successful synthesis of the amide bond between chitosan and rhamnolipid, which was also confirmed by Raman spectroscopy (Fig. 1H and I). It was speculated that HNK@RHL-COS NPs could break down in the low pH microenvironment of the infection site (pH 5.0–6.0) [41], as the amide bond formed by chitosan and rhamnolipid might be disrupted. Therefore, the *in vitro* release profiles of HNK@RHL-COS NPs were tested in different pH environments to assess their pH-responsive behavior. As expected, HNK@RHL-COS NPs showed rapid release, with over 40 % of HNK being released in the first 2 h at the acidic pH of 5.5, while only around 20 % was released at pH 7.4 (Fig. 1J). Both FT-IR and Raman spectroscopy showed the significantly reduced absorption peak of the amide bond of HNK@RHL-COS NPs after being incubated in an acidic environment for 48 h, confirming disruption of the chemical structure of HNK@RHL-COS NPs (Fig. 1H and I). These findings confirmed that HNK@RHL-COS NPs have notable pH-responsive properties.

### 3.2 In vitro antimicrobial activity

**Fig. 2:**
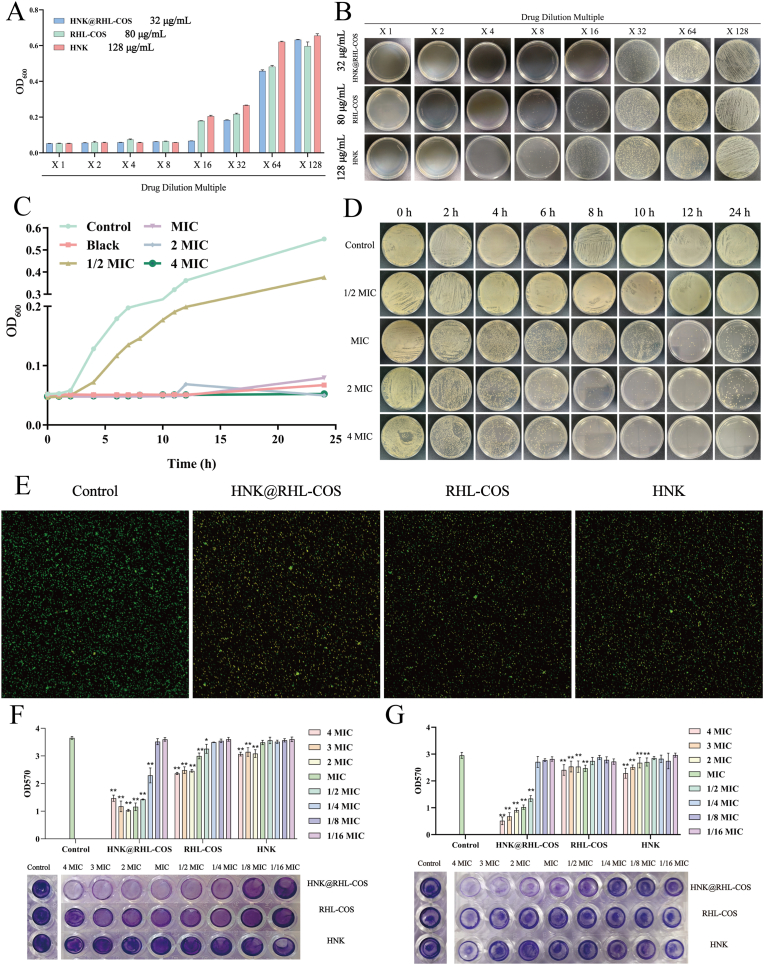
*In vitro* antimicrobial and anti-biofilm effects of HNK@RHL-COS NPs. (A) OD_600_ values of MRSA co-incubated with HNK@RHL-COS NPs, RHL-COS NPs and HNK at different concentrations for 12 h. (B) Plate counting images of MRSA after co-incubation with HNK@RHL-COS NPs, RHL-COS NPs and HNK at different concentrations for 12 h. (C, D) Plate counting images and OD_600_ values of MRSA incubated with different concentrations of HNK@RHL-COS NPs every 2 h. (E) Bacterial dead/live staining results. (F) The effect of HNK@RHL-COS NPs, RHL-COS NPs and HNK on the formation of MRSA biofilm. (G) The effect of HNK@RHL-COS NPs, RHL-COS NPs and HNK on the mature MRSA biofilm. Note: The values of MIC in HNK@RHL-COS NPs, RHL-COS NPs and HNK groups are 2 μg/mL, 10 μg/mL and 16 μg/mL, respectively. Statistical significance is denoted as ∗ *p* < 0.05, ∗∗*p* < 0.01, and ∗∗∗*p* < 0.001 vs the control group.

The antimicrobial activity was assessed by measuring OD_600_ values of MRSA co-incubated with HNK@RHL-COS, RHL-COS and HNK at different concentrations for 12 h, followed by colony-forming unit (CFU) counting on agar plates. The free drug HNK exhibited an MIC value of 16 μg/mL against MRSA. In contrast, the MIC values of HNK@RHL-COS NPs and RHL-COS NPs were found to be 2 μg/mL and 10 μg/mL, respectively. Similarly, the MBC values showed a comparable trend, with the values of HNK@RHL-COS NPs, RHL-COS NPs and HNK against MRSA being 3 μg/mL, 15 μg/mL and 25 μg/mL, respectively (Fig. 2A and B). These results indicated that HNK@RHL-COS NPs exhibit significantly enhanced antimicrobial activity, which may be attributed to the synergistic antimicrobial effect between HNK and chitosan.

Subsequently, the growth of MRSA was further monitored by measuring the OD_600_ values at various concentrations of HNK@RHL-COS, RHL-COS and HNK every 2-h intervals, followed by colony counting on agar plates. It was observed that the anti-MRSA efficacy of HNK@RHL-COS NPs at MIC, 2 × MIC and 4 × MIC concentrations increased with prolonged exposure time (Fig. 2C and D) [42]. Specifically, the HNK@RHL-COS NPs at MIC concentration showed the best antimicrobial effect within 12 h, while those at 2 × MIC and 4 × MIC concentrations achieved significant effects within 8 h, demonstrating a strong inhibitory effect of HNK@RHL-COS NPs on the growth of MRSA. Live/dead staining results revealed a predominance of dead bacteria (yellow) over live bacteria (green) in all treatment groups, with HNK@RHL-COS NPs exhibiting the best bactericidal effect against MRSA (Fig. 2E).

### 3.3 In vitro anti-biofilm

The formation of biofilms not only creates a protective barrier for bacteria, making it harder for drugs to penetrate, but also promotes the ongoing dispersal of planktonic bacteria, leading to repeated infections. Therefore, we investigated the antimicrobial effectiveness of HNK@RHL-COS NPs on MRSA biofilm. First, the impact of HNK@RHL-COS NPs on biofilm formation was assessed. Crystal violet-labeled biofilm was quantified by measuring the values of OD_570_. It was observed that the OD_570_ values for HNK@RHL-COS NPs at concentrations ranging from 1/4 × MIC to 4 × MIC were significantly lower than those of the control group (*p* < 0.05), indicating a substantial inhibitory effect on biofilm formation (Fig. 2F). In contrast, both the RHL-COS NPs group and the HNK group showed limited inhibitory effectiveness against MRSA biofilm formation, indicating a synergistic antibiofilm effect of HNK and RHL [10,12].

Thereafter, we further investigated the eradication efficacy of HNK@RHL-COS NPs on pre-formed biofilms. Our results demonstrated that HNK@RHL-COS NPs exhibited a significant dose-dependent inhibitory effect on mature biofilms within the concentration range of 1/2 × MIC to 4 × MIC, with OD_570_ values significantly lower than those of the control group (*p* < 0.05) (Fig. 2G). However, only at concentrations greater than the MIC was a slight anti-biofilm effect observed for the RHL-COS NPs group and the HNK group. Taken together, these results demonstrated that HNK@RHL-COS can not only inhibit biofilm formation but also eradicate existing biofilm, making it a potent alternative for treating clinical MRSA biofilm infections.

### 3.4 Destroying bacterial morphology

**Fig. 3:**
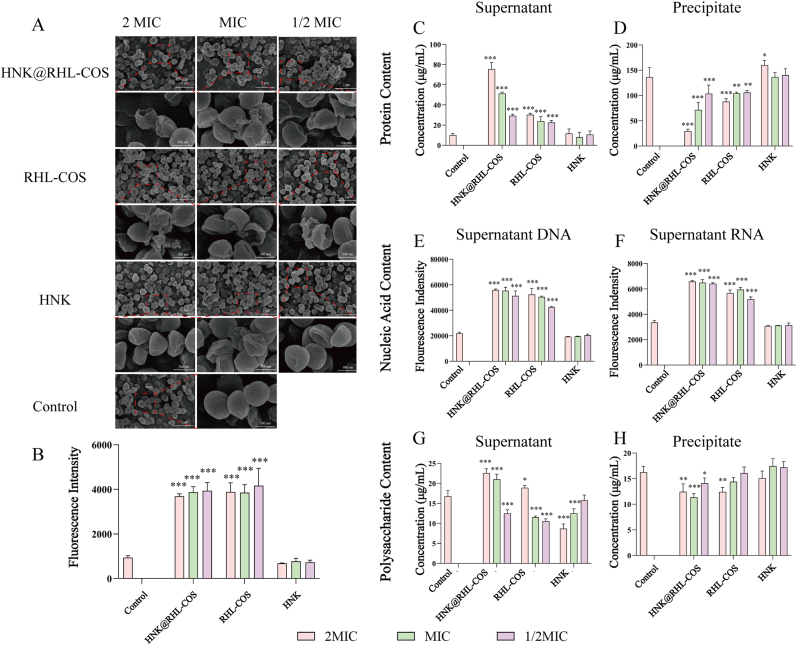
Antimicrobial mechanism of HNK@RHL-COS NPs. (A) SEM images of MRSA after treatment with HNK@RHL-COS NPs, RHL-COS NPs and HNK. (B) The PI fluorescence signal of MRSA after 12 h treatment with HNK@RHL-COS NPs, RHL-COS NPs and HNK (n = 3). Supernatant (C) and precipitate (D) protein content of MRSA after 12 h treatment with HNK@RHL-COS NPs, RHL-COS NPs and HNK (n = 3). Supernatant DNA (E) and RNA (F) content of MRSA after 12 h treatment with HNK@RHL-COS NPs, RHL-COS NPs and HNK (n = 3). Supernatant (G) and precipitate (H) polysaccharide content of MRSA after 12 h treatment with HNK@RHL-COS NPs, RHL-COS NPs and HNK (n = 3). Note: The values of MIC in HNK@RHL-COS NPs, RHL-COS NPs and HNK groups are 2 μg/mL, 10 μg/mL and 16 μg/mL, respectively. Statistical significance is denoted as ∗ *p* < 0.05, ∗∗*p* < 0.01, and ∗∗∗*p* < 0.001 vs the control group.

To further investigate the antimicrobial mechanism of HNK@RHL-COS NPs, we conducted scanning electron microscope (SEM) analysis on MRSA treated with different drugs [43]. MRSA in the control group maintained complete cell morphology and cell membrane structure (Fig. 3A). While after HNK@RHL-COS NPs treatment, significant destruction of the MRSA cell membrane was observed, as evidenced by contraction, collapse, rupture, and leakage of intracellular contents. Similar disruptions in cell membrane integrity were seen in MRSA treated with RHL-COS NPs at various concentrations. However, compared to the HNK@RHL-COS and RHL-COS groups, the cell membrane in the HNK group showed only mild shrinkage and collapse, with less rupture, indicating a weaker effect on the cell membrane.

### 3.5 Destruction of the MRSA cell membrane

Propidium iodide (PI) was used to assess the effect of NPs on the permeability of the MRSA cell membrane [44,45]. The fluorescence intensity in the HNK@RHL-COS and RHL-COS groups was significantly higher than that in the control and the HNK group, indicating greater membrane damage (Fig. 3B). In contrast, there was no significant difference in fluorescence intensity between the HNK group and the control group (*p* > 0.05), suggesting minimal membrane disruption. These results were consistent with SEM observations.

Furthermore, MRSA was treated with various groups, followed by centrifugation to quantify the protein content in both the supernatant and precipitate. Both the HNK@RHL-COS group and the RHL-COS group displayed significantly lower protein content in precipitates than in the supernatant, exhibiting a dose-dependent trend (Fig. 3C and D). These results indicated that both HNK@RHL-COS NPs and RHL-COS NPs effectively compromised bacterial membrane integrity, facilitating the efflux of intracellular proteins into the extracellular compartment. Notably, the damage caused by HNK@RHL-COS NPs was more severe. Next, to determine the leakage of MRSA nucleic acid, the DNA and RNA levels in the supernatant were quantified. Consistent with protein trends, the contents of DNA and RNA in the supernatant of the HNK@RHL-COS group were significantly higher than those in other groups (Fig. 3E and F) [46,47]. However, there was no obvious increase in the polysaccharide content in the supernatant, which is similar to that in the precipitate (Fig. 3G and H). The change of polysaccharides did not seem to have a corresponding trend, which may be due to the fact that at sublethal concentration, the membrane damage caused by NPs activated the cell wall repair process of bacteria, significantly up-regulated the autolysin activity, and promoted the efficient recovery of peptidoglycan degradation products for remodeling the cell wall, rather than releasing them to the extracellular. This active biological process resulted in a decrease in polysaccharide content in the supernatant [48,49]. This phenomenon not only strongly confirms that the core antibacterial mechanism of HNK@RHL-COS NPs is to destroy membrane integrity but also reveals that it can trigger a deadly stress response of bacteria, thus highlighting its superior antibacterial efficacy compared with other control groups.

### 3.6 Cell migration and *in vitro* anti-inflammatory

**Fig. 4:**
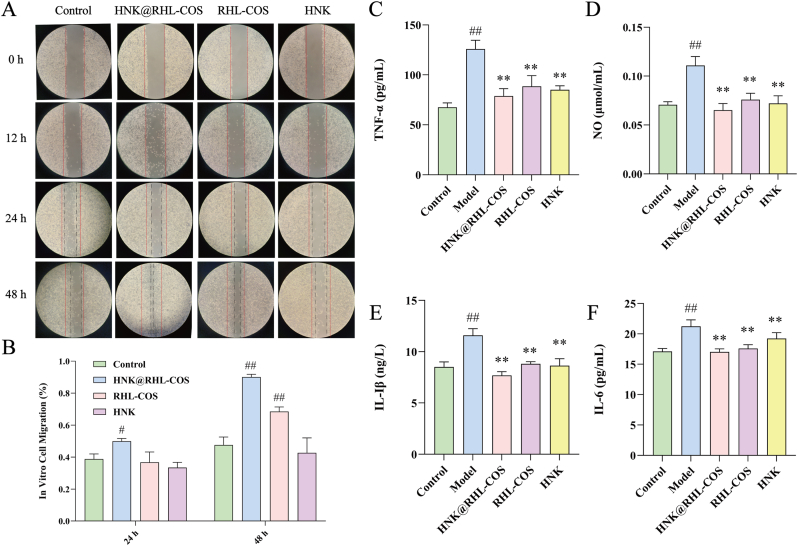
Cell migration and anti-inflammatory test results *in vitro*. (A, B) The migration image and the migration rate statistics of L929 cells after treatment with HNK@RHL-COS NPs, RHL-COS NPs and HNK (red line: original scratch, black line: migration distance) (n = 3). The levels of inflammatory factors TNF-α (C), NO (D), IL-1β (E) and IL-6 (F) in RAW264.7 cells after treatment with different drugs (n = 3). Statistical significance is denoted as ∗ *p* < 0.05 and ∗∗*p* < 0.01 vs the model group, and ^#^*p* < 0.05 and ^##^*p* < 0.01 vs the control group.

In order to explore the wound healing and anti-inflammatory effect of HNK@RHL-COS NPs, a cell migration assay and an *in vitro* anti-inflammatory assay were carried out in this study. Cell migration assay indicated that compared with the control group, the cell migration rate of the HNK@RHL-COS group was significantly increased at 24 h and 48 h (*P* < 0.05 and *P* < 0.01), indicating that HNK@RHL-COS NPs can effectively promote cell migration (Fig. 4A and B). On the other hand, HNK@RHL-COS NPs significantly reduced the levels of pro-inflammatory factors TNF-α, NO, IL-1β and IL-6 (*P* < 0.01), and the effect is better than that of the RHL-COS and HNK groups, suggesting that HNK@RHL-COS NPs have excellent anti-inflammatory activity *in vitro* (Fig. 4C–F).

### 3.7 Antimicrobial test *in vivo*

#### 3.7.1 Mouse skin infection model

**Fig. 5:**
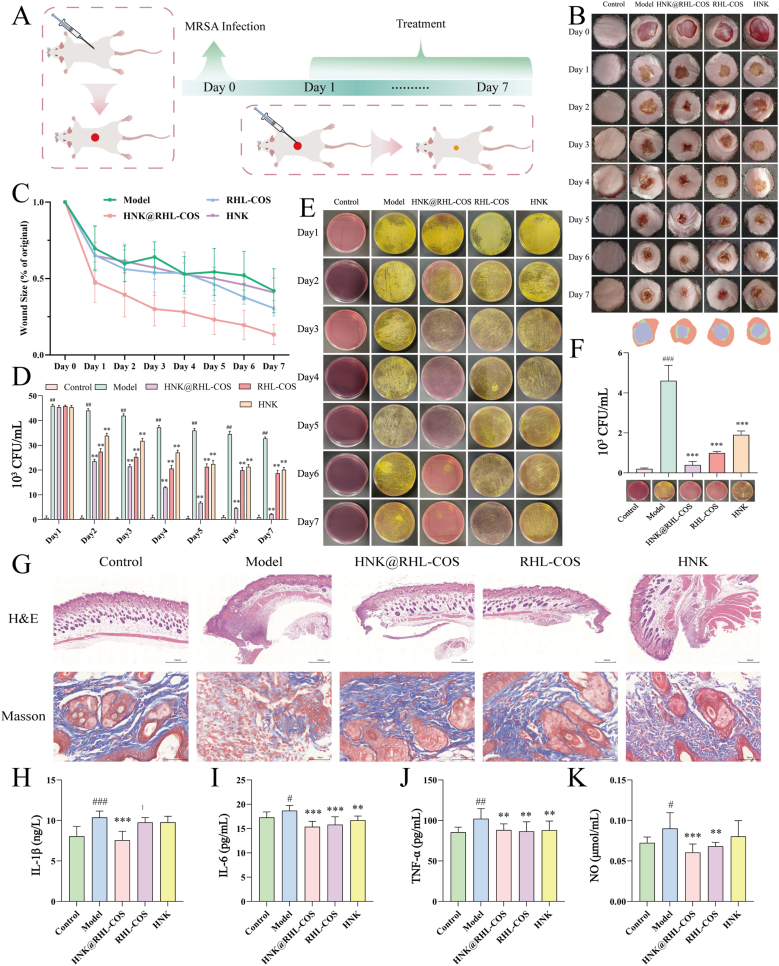
Effect of HNK@RHL-COS NPs on murine skin infection model. (A) Schematic diagram of the experimental process of *in vivo* wound infection treatment. (B) Photographs of wounds during treatment and wound superimpositions (orange for day 0, green for day 4, blue for day 7). (C) Statistical chart of changes in wound area during treatment (n = 6). (D, E) Plate counting image and statistics during treatment (n = 6). (F) Statistical chart of skin tissue bacteria after treatment (n = 6). (G) Masson staining and H&E staining of wound skin after treatment. IL-1β (H), IL-6(I), TNF-α (J) and NO (K) content after different treatments (n = 6). Statistical significance is denoted as ∗ *p* < 0.05, ∗∗*p* < 0.01, and ∗∗∗*p* < 0.001 vs the model group, and ^#^*p* < 0.05, ^##^*p* < 0.01, and ^###^*p* < 0.001 vs the control group.

Infectious skin diseases are a type of inflammatory skin lesion caused by pathogenic microorganisms [50]. Among these, skin infections caused by *S. aureus* and MRSA infections are common and challenging to heal quickly, owing to the associated skin inflammation induced by the released virulence factors such as proteases and δ toxins [[51], [52], [53], [54]]. Here, we initially developed a mouse skin infection model to study the *in vivo* antimicrobial activity of HNK@RHL-COS NPs (Fig. 5A). It was observed that the HNK@RHL-COS group had the fastest healing rate, with the wound area reducing to less than 50 % of the initial wound area by day 2 (Fig. 5B and C). In the RHL-COS group, the wound area reduced to 50 % of its original size by day 5. The significantly faster wound healing rate in the HNK@RHL-COS group compared to the HNK group may be due to its improved permeability and synergistic antimicrobial effect [55].

Additionally, the number of bacteria in the wound was measured using an *S. aureus* selective medium throughout the entire treatment period, since skin wounds are open and susceptible to contamination by various bacteria. It showed that the bacterial count in the model group was significantly higher than in the other groups during the whole treatment period (*p* < 0.05) (Fig. 5D and E). In the HNK@RHL-COS, RHL-COS and HNK groups, the bacteria count was significantly lower than that in the model group (*p* < 0.05). Notably, the bacterial count in HNK@RHL-COS NPs decreased most rapidly, and after seven days of treatment, the HNK@RHL-COS group showed the lowest bacterial load among all groups (Fig. 5F). These results demonstrate that HNK@RHL-COS NPs can significantly eliminate bacteria at the infection site, which is beneficial to wound healing.

Wound healing is primarily assessed by investigating the collagen content within the wound [56]. Therefore, Masson staining was performed on the skin at the wound site. In the model group, there was less collagen content at the wound site, and its arrangement was notably loose (Fig. 5G). The collagen in the HNK@RHL-COS group appeared darker in color, more abundant, better aligned and denser. This suggests that skin structural reconstruction was improved in the HNK@RHL-COS group. H&E staining also showed significant infiltration of inflammatory cells in the model group, whereas the treatment groups effectively reduced inflammatory cell infiltration at the infection site (Fig. 5G). In *S. aureus*-infected skin wounds, bacterial exposure attracts eosinophils to the affected skin, thereby promoting inflammation [57]. Therefore, ELISA was used to determine the levels of inflammatory factors in the serum of mice. The results showed that the levels of IL-6, IL-1β, TNF-α and NO in the model group were significantly higher than in the control group (*p* < 0.05). After HNK@RHL-COS treatment, the levels of inflammatory factors were down-regulated (*p* < 0.001) (Fig. 5H–K). Collectively, these results demonstrate that HNK@RHL-COS NPs effectively alleviated the inflammation induced by MRSA infection and promoted wound healing.

#### 3.7.2 Bacterial peritonitis

**Fig. 6:**
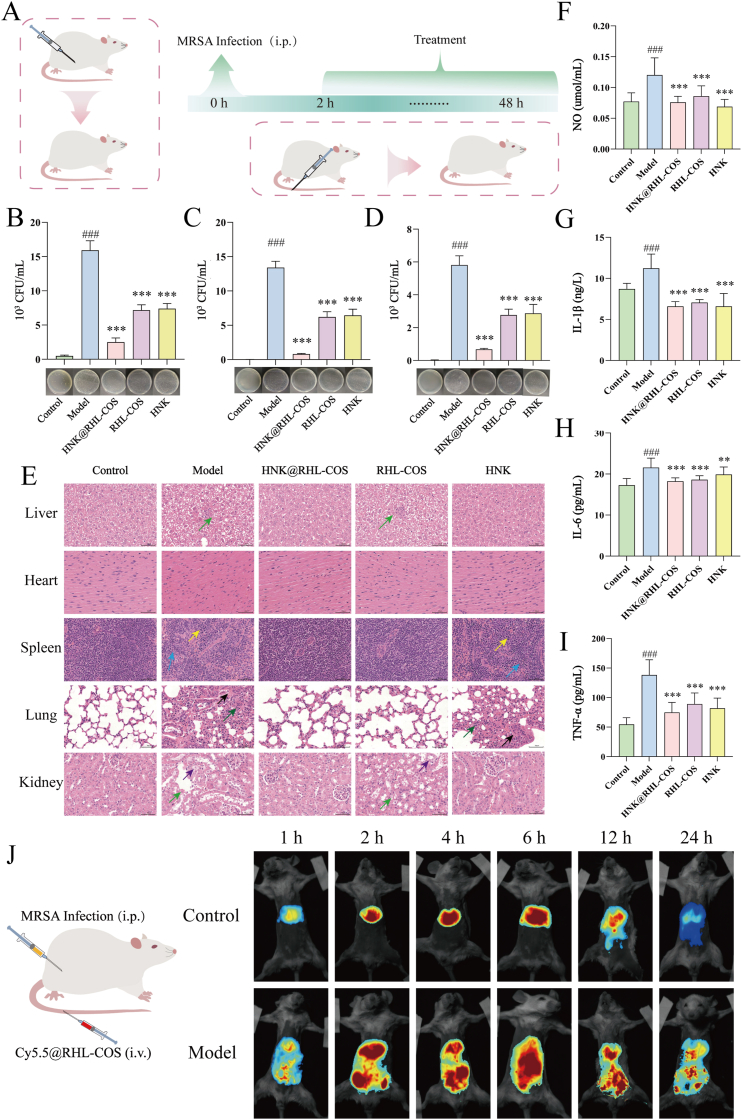
Effect of HNK@RHL-COS NPs on murine bacterial peritonitis. (A) Schematic diagram of the experimental process of *in vivo* bacterial peritonitis. The total bacterial load in the ascites (B), extracellular bacteria (C), and intracellular bacteria (D) of each group (n = 6). (E) H&E staining of liver, heart, spleen, lung and kidney (green: cell necrosis; yellow: increased erythroid cells; blue: granulocyte cells increased; black: fibroblast hyperplasia; dark green: alveolar epithelial cell hyperplasia; purple: cellular degeneration). NO (F), IL-1β (G), IL-6 (H) and TNF-α (I) levels after different treatments (n = 6). (J) *In vivo* imaging at different times after Cy5.5@RHL-COS injection. Statistical significance is denoted as ∗ *p* < 0.05, ∗∗*p* < 0.01, and ∗∗∗*p* < 0.001 vs the model group and ^#^*p* < 0.05, ^##^*p* < 0.01, and ^###^*p* < 0.001 vs the control group.

Bacterial peritonitis, caused by bacteria entering the ascitic fluid, has a very high mortality rate [58,59]. Therefore, we subsequently evaluated the therapeutic efficacy of HNK@RHL-COS NPs using a bacterial peritonitis model (Fig. 6A). After treatment, the total bacterial load, extracellular bacteria and intracellular bacteria in the ascitic fluid of each group were significantly reduced, markedly lower than those in the model group (*p* < 0.001) (Fig. 6B, C and D), with HNK@RHL-COS NPs exhibiting the most superior antimicrobial efficacy.

Furthermore, H&E staining was performed on the organs of each group of mice (Fig. 6E). In the model and RHL-COS groups, some liver cells exhibited necrosis, and a small number of renal tubular epithelial cells showed signs of degeneration and necrosis. Moreover, both the model group and HNK group displayed elevated splenic erythrocyte and granulocyte counts, increased alveolar epithelial cells, and enhanced pulmonary interstitial fibrosis. In contrast, no noticeable pathological changes were observed in the HNK@RHL-COS and control groups. The inflammatory factors (IL-6, IL-1β, TNF-α, and NO) in the model group were markedly elevated in the control group (*p* < 0.001), whereas HNK@RHL-COS, RHL-COS and HNK group treatment significantly reduced these factors (*p* < 0.001) (Fig. 6F–I). These results demonstrated that HNK@RHL-COS NPs effectively attenuated inflammation and associated inflammatory damage in bacterial peritonitis mice.

Encouraged by the remarkable effects of HNK@RHL-COS NPs on the bacterial peritonitis, we then evaluated the biodistribution of NPs after *i.v.* injection using Cy5.5@RHL-COS NPs. The fluorescence signal in the control group was primarily concentrated in the liver region, while distinct drug fluorescence distribution was observed in both the abdominal cavity and the liver in the model group, with the fluorescence intensity peaking at 6 h (Fig. 6J). These results indicate that HNK@RHL-COS NPs have an ideal targeted effect against MRSA infection.

### 3.8 Biocompatibility evaluation

**Fig. 7:**
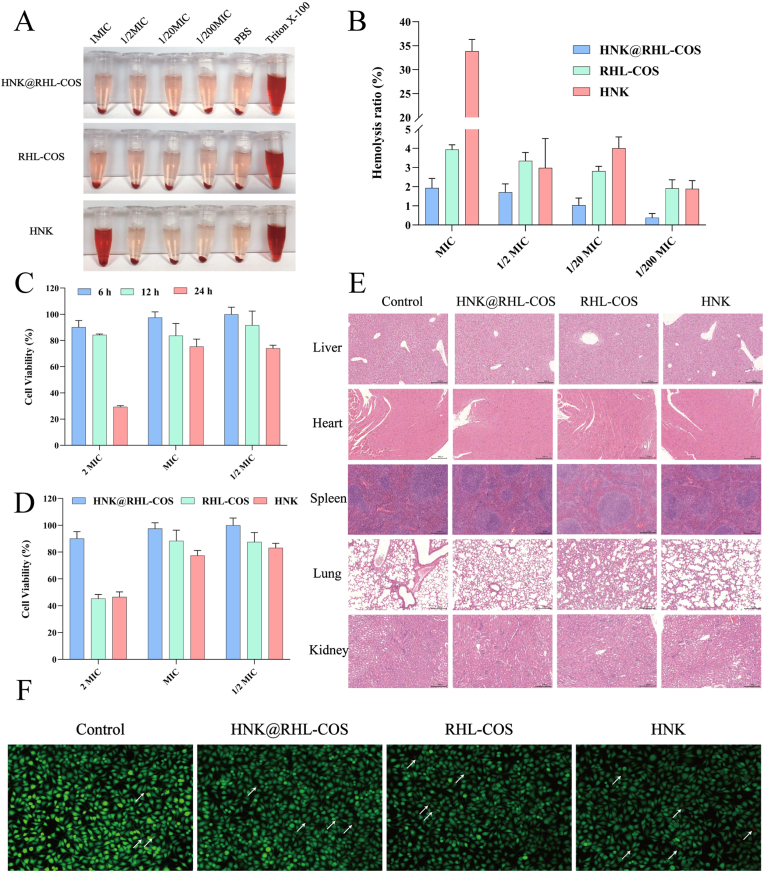
Biocompatibility evaluation. (A, B) Hemolysis assay results of HNK@RHL-COS NPs, RHL-COS NPs and HNK (n = 3). (C) L929 cell viability after treating with different concentrations of HNK@RHL-COS NPs (n = 5). (D) The survival rate of L929 cells treated with HNK@RHL-COS NPs, RHL-COS NPs and HNK for 12 h (n = 5). (E) H&E staining of liver, heart, spleen, lung and kidney (n = 6). (F) Live/dead staining results (White arrows are red-stained dead cells) (n = 3).

Safety is a crucial factor in determining whether HNK@RHL-COS NPs are suitable for further development. Therefore, we performed cytotoxicity, hemolysis tests, and *in vivo* toxicity assessments to evaluate the compatibility of HNK@RHL-COS NPs [60,61]. It was observed that the hemolysis rate of HNK@RHL-COS NPs was less than 5 % at a concentration of MIC, whereas the HNK group achieved a hemolysis rate below 5 % at a concentration of 1/2 × MIC, indicating that HNK@RHL-COS NPs have good biocompatibility (Fig. 7A and B). The CCK-8 assay showed that the corresponding cell viability of L929 exceeded 80 % at HNK@RHL-COS NPs concentrations below 2 × MIC, while HNK and RHL-COS NPs displayed a cell viability of less than 50 % after treating with a 2 × MIC concentration of the corresponding drug (Fig. 7C and D). Overall, the hemolysis rate and cytotoxicity data indicate that HNK@RHL-COS NPs demonstrate greater safety and biocompatibility *in vitro* than HNK and RHL-COS NPs, making them suitable for further investigation.

Subsequently, the *in vivo* toxicity of HNK@RHL-COS NPs was evaluated using a mouse model. After drug treatment, the diet and water intake of mice in each group were comparable to those of the control group, indicating that HNK@RHL-COS NPs did not influence the feeding behavior of the mice (Fig. S3A and B). No significant change was observed in the organ index of each group (*p* > 0.05), and the body weight changes remained within a reasonable range without significant variation in each group (*p* > 0.05) (Fig. S3C and D). In addition, blood routine analysis revealed no significant differences between mice treated with HNK@RHL-COS NPs, RHL-COS NPs and HNK compared to the control group (*p* > 0.05), indicating excellent biocompatibility of HNK@RHL-COS NPs (Fig. S4). H&E staining of mouse organs showed that there were no significant pathological changes in the heart, liver, lung, kidney, and spleen of mice after treatment with HNK@RHL-COS NPs, RHL-COS NPs and HNK (Fig. 7E). Finally, live/dead staining of L929 cells treated with different concentrations of HNK@RHL-COS revealed that there was no obvious effect on the proliferation and growth of L929 cells (Fig. 7F). Collectively, these results demonstrated that HNK@RHL-COS NPs exhibit excellent biocompatibility *in vitro* and *in vivo*, supporting the further development and application.

## 4 Conclusion

In this study, we developed HNK-based nanoparticles for treating MRSA infections. HNK@RHL-COS NPs exhibited good pH-responsive properties and stability, demonstrating strong antimicrobial activity and the ability to disrupt MRSA biofilm both *in vivo* and *in vitro*. The antimicrobial mechanism of HNK@RHL-COS NPs was examined and revealed that it primarily involves destroying the MRSA cell membrane. HNK@RHL-COS NPs significantly accelerated wound healing and reduced inflammation at the infection site in a mouse infection model. Moreover, the combined use of HNK and COS significantly decreased toxicity and expanded their potential applications. This research presents a safe, effective and promising antibiotic-free antimicrobial alternative for clinically treating MRSA infections and minimizing the development of bacterial resistance.

## CRediT authorship contribution statement

**Shouli Yi:** Writing – review & editing, Writing – original draft, Visualization, Software, Methodology, Investigation, Formal analysis, Data curation, Conceptualization. **Hongjuan Zhang:** Writing – review & editing, Writing – original draft, Validation, Supervision, Resources, Project administration, Methodology, Investigation, Conceptualization. **Zhen Yang:** Visualization, Investigation, Formal analysis. **Di Wu:** Formal analysis, Data curation, Conceptualization. **Dan Shao:** Visualization, Formal analysis. **Jiongjie He:** Formal analysis, Conceptualization. **Yu Liu:** Investigation, Formal analysis. **Baocheng Hao:** Writing – review & editing, Supervision, Software, Methodology, Funding acquisition. **Shengyi Wang:** Writing – review & editing, Supervision, Software, Resources, Methodology, Funding acquisition.

## Declaration of competing interest

Shouli Yi declares that she has no conflicts of interest. Hongjuan Zhang declares that she has no conflicts of interest. Zhen Yang declares that she has no conflicts of interest. Di Wu declares that she has no conflicts of interest. Dan Shao declares that she has no conflicts of interest. Jiongjie He declares that he has no conflicts of interest. Yu Liu declares that he has no conflicts of interest. Baocheng Hao declares that he has no conflicts of interest. Shengyi Wang declares that he has no conflicts of interest.

## Data Availability

Data will be made available on request.
