# Supplementary material for: Honokiol-loaded nanoparticles for targeted bacterial eradication and treatment of MRSA infection
Source: Mater Today Bio. 2025 Nov 17;35:102562. doi: 10.1016/j.mtbio.2025.102562 (PMC12676111; doi:10.1016/j.mtbio.2025.102562)
Supplement: Multimedia component 1 [file mmc1.docx]

**Honokiol-loaded nanoparticles for targeted bacterial eradication and treatment of MRSA infection**

**Running title:** Honokiol NPs for targeted MRSA therapy.

Shouli Yi ^a, b, #^, Hongjuan Zhang ^a, #^, Zhen Yang ^a^, Di Wu ^a^, Dan Shao ^a^, Jiongjie He ^a^, Yu Liu ^a^, Baocheng Hao ^a, *^, Shengyi Wang ^a, *^

^a^ Key Laboratory of New Animal Drug Project, Gansu Province/Key Laboratory of Veterinary Pharmaceutical Development, Ministry of Agriculture and Rural Affairs/Lanzhou Institute of Husbandry and Pharmaceutical Sciences of Chinese Academy of Agricultural Sciences, Lanzhou 730050, P.R. China

^b^ College of Animal Science and Technology, Guangxi University, Nanning 530005, P.R. China.

^#^These authors have contributed equally to this work and share first authorship.

*** Correspondence:**

**Shengyi Wang,** Lanzhou Institute of Husbandry and Pharmaceutical Sciences of Chinese Academy of Agricultural Sciences, Lanzhou, 730050, P.R. China.

Tel: +86 0931 2115286, E-mail: [wangshengyi@caas.cn](mailto:wangshengyi@caas.cn) (S. Wang).

**Baocheng Hao,** Lanzhou Institute of Husbandry and Pharmaceutical Sciences of Chinese Academy of Agricultural Sciences, Lanzhou, 730050, P.R. China.

Tel: +86 0931 2115286, E-mail: [haobaocheng@caas.cn](mailto:haobaocheng@caas.cn) (B. Hao).

**
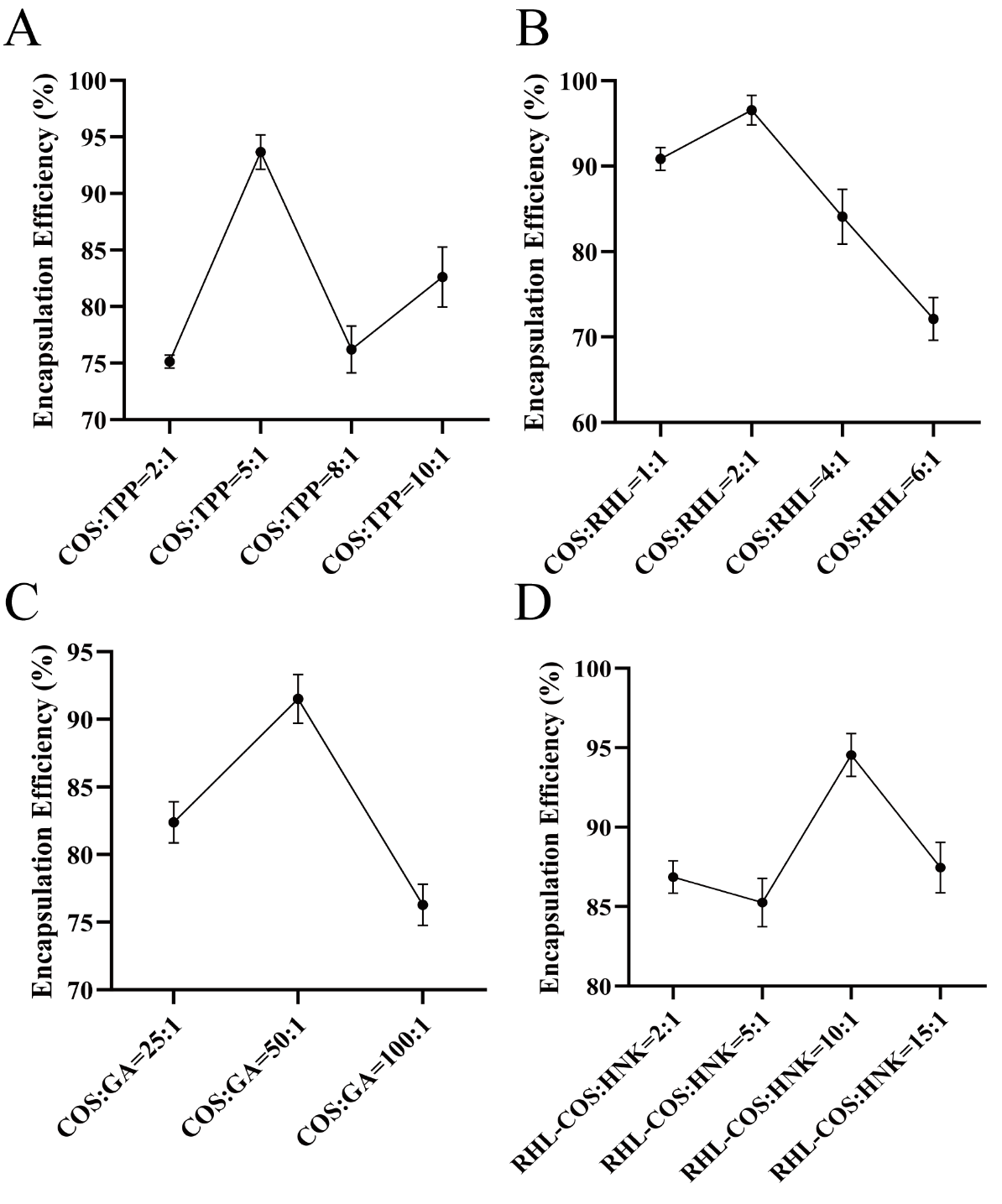
**

**Fig. S1.** Prescription screening results of HNK@RHL-COS NPs based on the encapsulation efficiency. The effect of the ratios of chitosan and sodium tripolyphosphate (A), chitosan and rhamnolipid (B), chitosan and glutaraldehyde (C), and RHL-COS NPs and honokiol (D) on the encapsulation efficiency of HNK@RHL-COS NPs.

**
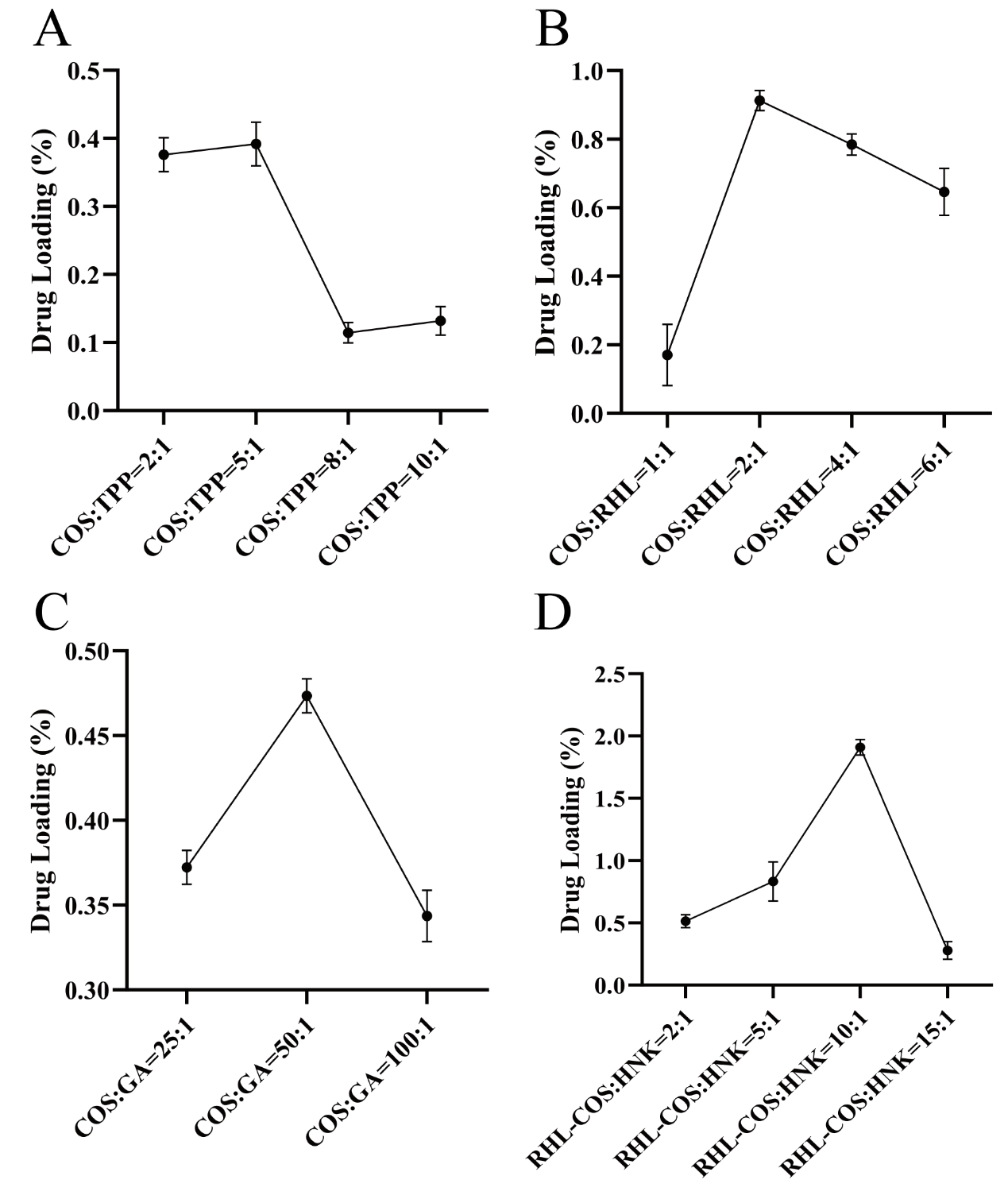
Fig. S2.** Prescription screening results of HNK@RHL-COS NPs based on the drug loading. The effect of the ratios of chitosan and sodium tripolyphosphate (A), chitosan and rhamnolipid (B), chitosan and glutaraldehyde (C), and RHL-COS NPs and honokiol (D) on the drug loading of HNK@RHL-COS NPs.

**
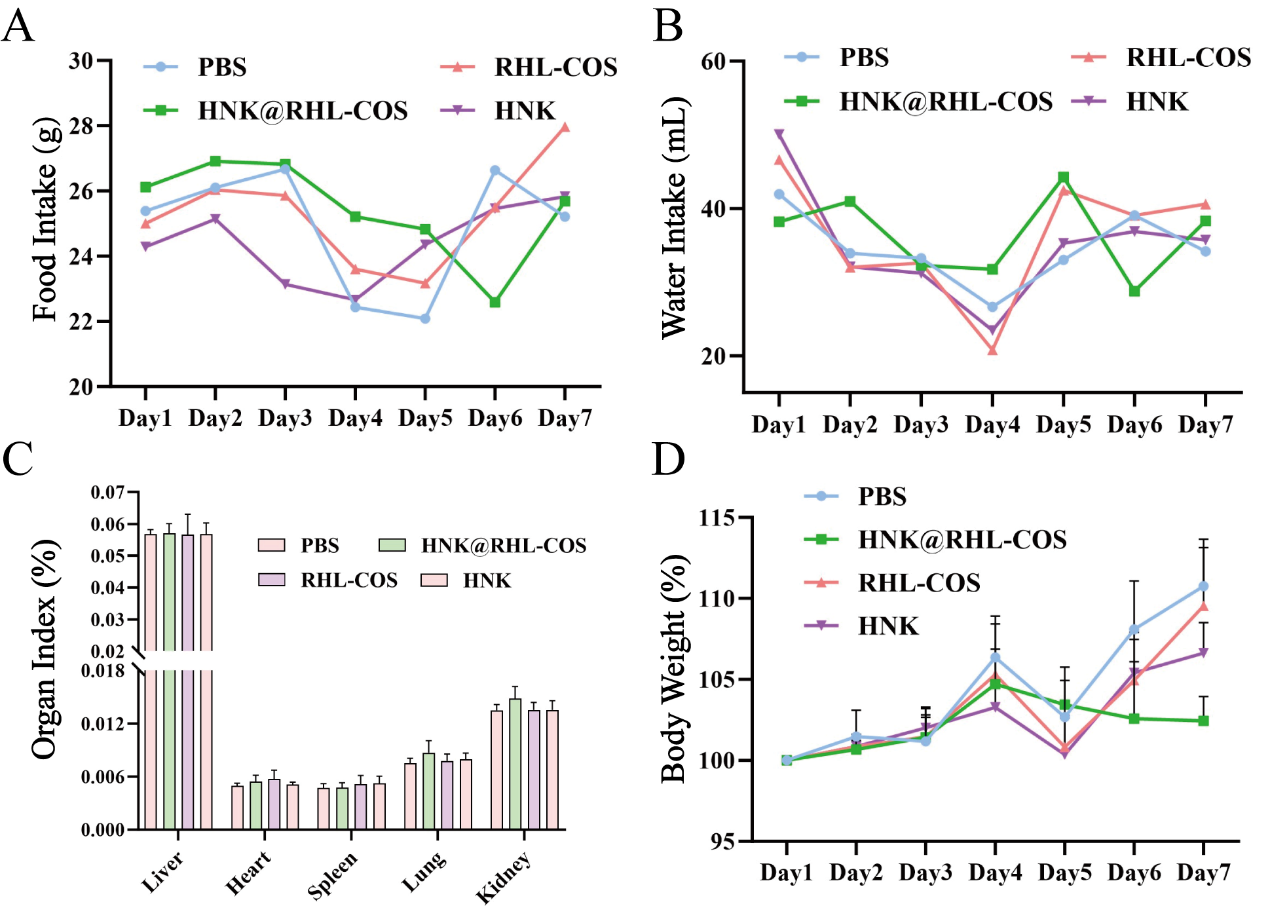
**

**Fig. S3.** Safety experimental mouse indicators. (A) Food intake of mice during administration. (B) Water intake of mice during administration. (C) Changes in organ index of mice after administration. (D) Changes in body weight of mice during administration (n = 6).

**
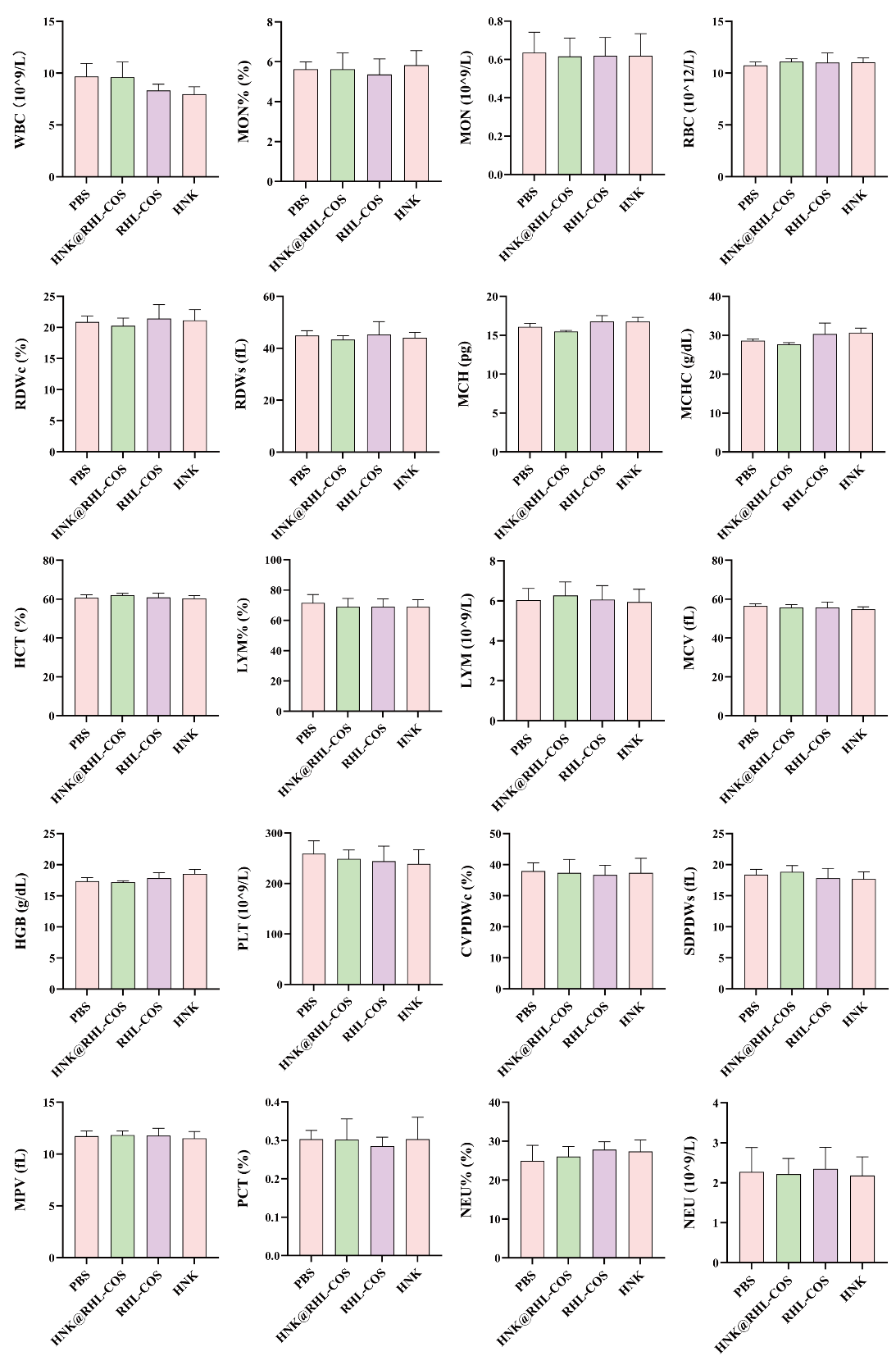
**

**Fig. S4.** Blood routine index of mice (n = 6).
